# Exploring the relationship between obstetric violence, postpartum depression, and breastfeeding through structural equation modelling: evidence from 2015 Pelotas birth cohort

**DOI:** 10.1186/s12889-025-22851-9

**Published:** 2025-08-16

**Authors:** Marilia Arndt Mesenburg, Emanuele Souza Marques, Tatiana Henriques Leite, Maria do Carmo Leal, Mariangela Freitas Silveira

**Affiliations:** 1 Present Address: National Institute of Women’s, Children’s, and Adolescents’ Health Fernandes Figueira, Oswald Cruz Foundation, Rio de Janeiro, Brazil; 2 Present Address: Pontifical Catholic University of Ecuador, Quito, Ecuador; 3 Institute of Social Medicine Hesio Cordeiro, Rio de Janeiro State University, Rio de Janeiro, Brazil; 4 National School of Public Health, Oswaldo Cruz Foundation, Rio de Janeiro, Brazil; 5 Postgraduate Program in Epidemiology, Federal University of Pelotas, Pelotas, Brazil

**Keywords:** Obstetric violence, Breastfeeding, postpartum depression, Structural equation modelling, women health, global health

## Abstract

**Background:**

Obstetric violence is a gender-based violence against women perpetrated by health professionals during pregnancy and childbirth and presents negative consequences for women and newborns. Our objective was to evaluate the effect of obstetric violence on breastfeeding and the role of postpartum depression as a mediator.

**Methods:**

We analysed data from 4275 women and their newborns enrolled in the Pelotas 2015 Birth Cohort by type of delivery (vaginal and c-section). We performed structural equation modelling. The exposure to obstetric violence during childbirth was a latent variable, and the outcome was the duration of breastfeeding. We evaluate postpartum depression as a possible mediator of that relationship.

**Results:**

Women who suffered obstetric violence presented a higher probability of interrupting breastfeeding earlier than those who did not. This effect was observed only among women who had vaginal delivery (-0.224; *p* value: 0.017). We did not find an effect of obstetric violence on breastfeeding among women who had c-section deliveries. The effect of postpartum depression as a mediator on the relationship between obstetric violence and breastfeeding was not demonstrated. The total effect (direct + indirect effect) among women who had vaginal delivery was − 0.223, *p* value: 0.016. The total effect for the c-section group did not attain statistical significance.

**Conclusions:**

The occurrence of obstetric violence decreases the duration of breastfeeding among women who have vaginal births. We were unable to demonstrate the same effect among the c-section group. We did not identify postpartum depression as a mediator of the association studied. This is the first study to evaluate the role of postpartum depression as a mediator of the association between obstetric violence and breastfeeding and the second to evaluate this relationship. Further studies must be conducted to better understand this subject.

## Introduction

Obstetric violence is a form of gender-based violence against women. Over time different terms have been used to describe this violation of women’s human rights, such as mistreatment, disrespect and abuse during pregnancy and childbirth [1–3]. The term “obstetric violence” was created in Venezuela (2007) by social movements for women’s reproductive rights and is the most used in Latin America and the Caribbean. Since 2022 there has been an expressive increase in the number of European and North American countries that began using the name [4–7]. The term obstetric violence describes the phenomenon most properly since it encompasses pregnancy, puerperium, and abortion, which have been excluded from other definitions. Obstetric violence also incorporates all the aspects of this gender violence, including the place where occurs; perpetrators (health professionals’ majority); procedures performed excessively, without consent or clinical indication, and non-based on scientific evidence. Finally, to use the term obstetric violence is to provide support to victims and activists in mitigating this type of violence [8].

In terms of definition, despite there being no consensus in the field, the proposal of Bohren et al. [2] has been the most accepted by the scientific community. The authors divided the definition into seven dimensions: (I) physical violence, (II) sexual violence, (III) verbal violence, (IV) stigma and discrimination, (V) failure to provide qualified care, (VI) failure to communicate with the healthcare team, and (VII) inadequacy of the health system. The discussion of the definition, dimensions, and acts to be included in each one, remains a challenge since it includes different perspectives from diverse cultural contexts. So far, there is no validated instrument to evaluate the topic.

This type of violence has been recognized by the WHO as a public health problem [3, 9, 10]. However, several studies conducted in different settings have indicated high occurrence independent of cultural or social contexts or country income level [5, 11–16]. For instance, a study carried out in Spain comprising 17,541 women showed that 38.5% of them perceived having suffered obstetric violence [5]. A similar pattern was found in another wide national survey conducted in the Netherlands including 12,239 women, of which 36.3% reported having experienced some situation of disrespect or abuse between 2018 and 2019 [13, 14]. Likewise, findings from a nationally representative study conducted in Brazil between 2011 and 2012 involving approximately 20,500 women showed that 44% of them reported having experienced obstetric violence [15].

The number of studies focused on assessing the prevalence of obstetric violence has increased notably in recent years. However, the literature concerning its consequences for women and/or their children remains limited. Most investigations on this topic concentrate on evaluating the effect of obstetric violence on women’s mental health. Recent studies have evidenced a positive association between obstetric violence and postpartum depression, as well as posttraumatic stress disorder [15, 17]. The influence of obstetric violence during childbirth on the utilization of postpartum healthcare services was studied in Brazil. The researchers found that women who had experienced obstetric violence delayed or reduced the use of such services, both for themselves and their newborns, compared to those who had not experienced this type of violence [18].

Recently, a pioneering study brought to light the relationship between obstetric violence and breastfeeding. This was the first epidemiological study to investigate the subject. Leite et al. (2023) analysed data from a large national study conducted between 2011 and 2012 in Brazil, including approximately 20,500 women recruited at maternity during hospitalization for delivery and interviewed by phone 43–180 days later. The results demonstrated a negative effect of obstetric violence on breastfeeding [19].

The benefits of breastfeeding lead the WHO to recommend “mothers worldwide to exclusively breastfeed infants for the child’s first six months to achieve optimal growth, development, and health. Thereafter, they should be given appropriate complementary foods and continue breastfeeding up to the age of two years or beyond” [20]. The benefits for children who are adequately breastfed include lower morbidity and mortality rates [21], fewer dental caries occurrence [22], and higher intelligence coefficients [23] compared to those who are either not breastfed or breastfed for shorter durations. For women, breastfeeding benefits include the prevention of breast cancer, a risk reduction of diabetes and ovarian cancer, and improved birth spacing [24]. It is estimated that near universal breastfeeding levels could prevent approximately 823,000 child deaths and 20,000 breast cancer-related deaths annually [21].

Given the benefits of breastfeeding, the magnitude of obstetric violence worldwide, and the innovative evidence on the connection between both, advancing in understanding this relationship holds great interest to world public health. Unravelling how this effect occurs is fundamental [19]. The study objective was to investigate the effect of obstetric violence on breastfeeding and the potential role of postpartum depression in this association. We hypothesize that postpartum depression acts as a mediator of the relationship between obstetric violence and breastfeeding.

## Methods

### Design and participants

The sample comprised participants from the 2015 Pelotas Birth Cohort Study, a population-based cohort of all live births from mothers living in the urban area of Pelotas city, a median-sized city in southern Brazil. All women residents in the urban area of the city with confirmed pregnancy estimated delivery date in the year 2015 were invited to take part in the antenatal follow-up of the cohort. Eligible pregnant women were recruited from antenatal care health services, and face-to-face interviews were conducted using structured questionnaires. Information on depression during gestation was assessed mid-pregnancy (16–22 weeks).

**Fig. 1:**
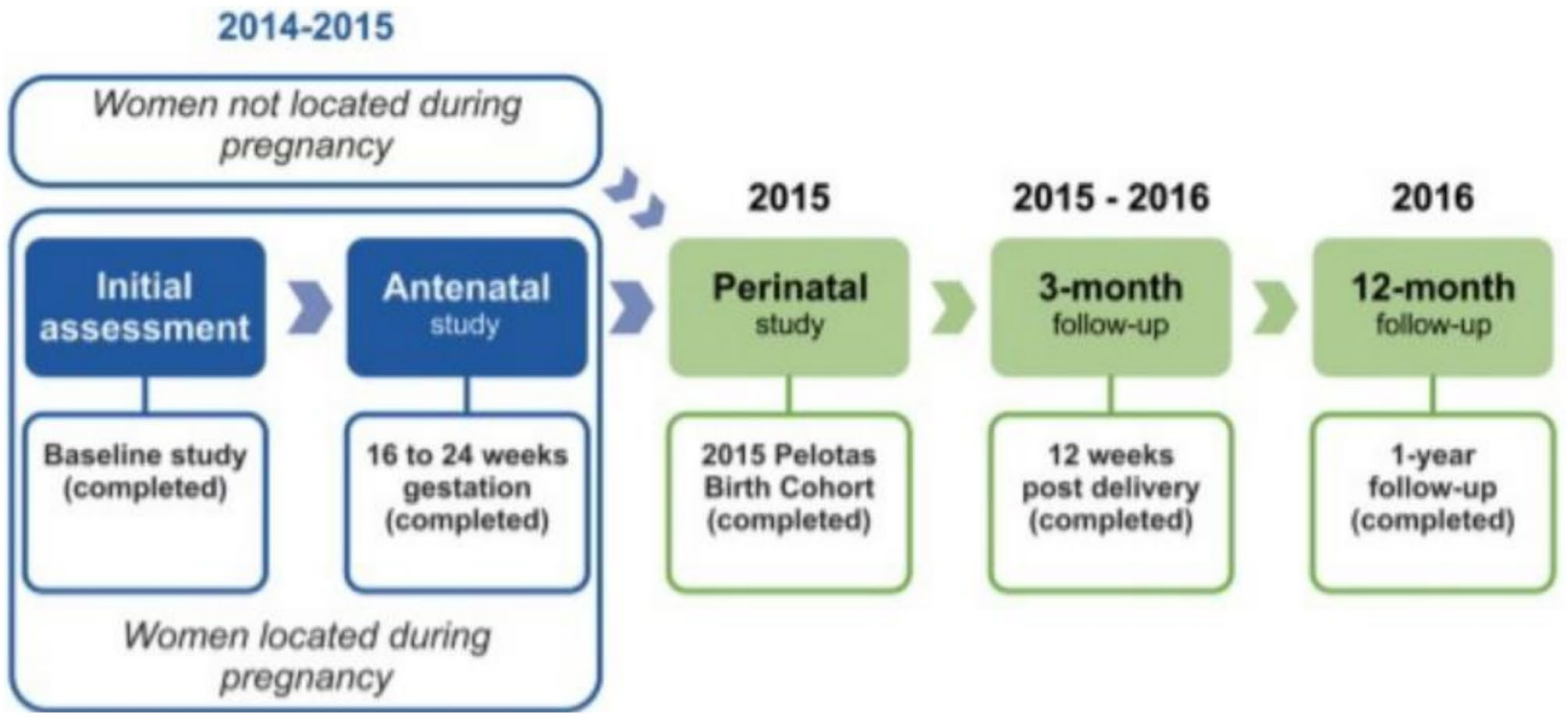
2015 Pelotas Birth Cohort recruitment and follow-up schedule adapted from Hallal et al. [17]

From January 1 to December 31, 2015, maternity hospitals were visited daily, and all births were identified (over 99% of births in the city). In all, 4,333 live births were detected. Mothers of 4275 newborns (response rate 98.7%) agreed to enroll in the study and composed the final sample. Within 48 h after delivery, they replied to a face-to-face interview. At the age of three months, the children were visited at home by interviewers, and their mothers answered another questionnaire. A total of 4,110 3-month follow-up interviews were conducted (response rate 97.2%), of which 4,087 (95.6%) were carried out with the biological mothers. Figure 1 shows the 2015 Pelotas Birth Cohort recruitment and follow-up schedule. For the present study, we excluded from the analyses participants with conditions that could preclude breastfeeding or make it difficult, including women whose newborn was admitted to the intensive care unit, those who had twins, those who had a preterm delivery (< 37 weeks) and women diagnosed as being positive for human immunodeficiency virus (HIV). The final sample comprises data from 3,598 biological mothers. All information was collected through structured questionnaires applied face-to-face by interviewers who had completed undergraduate training. The interviewers participated in theoretical and practical training with, on average, two weeks of duration. Details about the methodology were previously published [25].

### Measurements

#### Breastfeeding - outcome

This study explored breastfeeding in two different ways: (i) *breastfeeding duration* in months and (ii) attempt to breastfeed in the maternity unit. The first one was collected during the 12-month follow-up by asking “Until what age was the child breastfed?”. The range of responses varied from zero to 12 months. Due to the small number of children breastfed after eight months, we joined those breastfed for nine or more months into a unique category. Attempts to breastfeed while in the maternity ward were collected through the question “Have you already put the baby to the breast?” (response options “yes” or “no”).

#### Obstetric violence – exposure

Self-reported experience of obstetric violence was assessed during household interviews with the biological mothers three months after delivery. Due to the lack of consensus in the literature concerning its definition and the absence of validated instruments, we opted to operationalize obstetric violence as a latent variable composed of four items as described below.

Information on verbal abuse, physical abuse, negligence/denial of care, and undesired/non-consented procedures during hospitalization for childbirth were measured using the following questions: **Verbal abuse**: “Has any professional been rude to you, cursed you or yelled at you, humiliated you or threatened not to assist you?”; **Physical abuse**: “Has any professional ever pushed, hurt, beat, or held yourself strongly or conducted any examinations rudely or disrespectfully?”; **Negligence/denial of care**: “Has any professional refused to give you anything that you asked for, such as water or painkillers?”; **Undesired/Non-consented procedures**: “Has any professional ever conducted any procedure against your will, without explaining the need to conduct it, such as episiotomy or medication to induce labour?” To ensure that women would respond about events that occurred during the hospitalization for her most recent childbirth – subject matter of this study, the following introduction was included at the beginning of the obstetric violence questions block: “Now, let’s talk about the care you received during your hospitalization for the birth of your baby. To answer the next questions, consider what happened from the moment you arrived at the maternity ward until the moment you were discharged”.

#### Postpartum depression – mediator

Symptoms of postpartum depression were evaluated during face-to-face interviews conducted using the Edinburgh Postnatal Depression Scale (EPDS) three months after delivery. This scale comprises ten items, each scored on a 4-point Likert scale (ranging from 0 to 3), addressing common depressive symptoms experienced during the preceding week. A composite score is obtained by summing all item scores, resulting in a range from 0 (indicative of the absence of depressive symptoms) to 30 (denoting the highest score) [18]. A cut-off point of ≥ 13 points on the scale was employed to indicate the presence of at least moderate postpartum depression. Considering clinical interviews as the gold standard, this cut-off point has demonstrated a sensitivity of 59.6% (95% CI: 49.5–69.1) and specificity of 88.3% (95% CI: 83.9–91.9) for diagnosing depression [26].

The Cronbach alpha in this study was 0.796.

#### Potential confounders

**Fig. 2:**
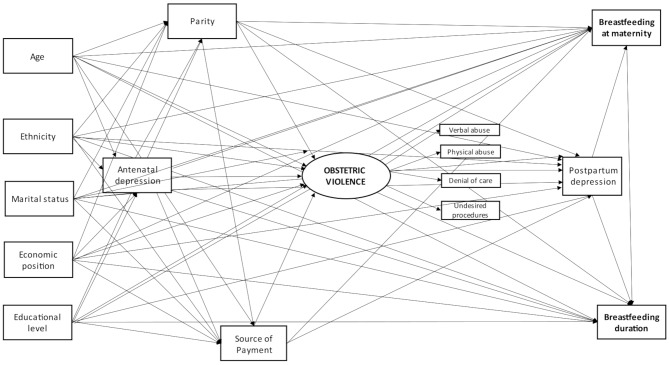
Theoretical-operational model concerning the relationship between obstetric violence and breastfeeding. This model was similar for women who underwent a vaginal and a c-section delivery

According to the theoretical-operational model presented in Fig. 2, at the first level, we included as potential confounders woman’s age in years; marital status (living with a partner or not); woman’s educational level; socioeconomic position, and self-reported ethnicity. The women’s educational level was collected in completed years and categorized into 0 to 4, 5 to 8, 9 to 11 and 12 years or more. Socioeconomic position was evaluated according to the Brazilian Economic Classification Criteria (ABEP) on 01/01/2015 (available at http://www.abep.org/criterio-brasil) and categorized into four economic groups: A (richest), B, C, and D/E (poorest). Self-reported ethnicity information was collected following the definition of the Brazilian Institute of Geography and Statistics (categorized as black, light-skinned black, white, Asian, and Indigenous) and categorized as white or non-white due to the small number of Indigenous and Asians in the sample. At the second level, we included antenatal depression measured by applying the EPDS between the 16th and 24th weeks of pregnancy. Positive screening for antenatal depression was defined as EPDS ≥ 10, which is the recommended cut-off for screening in the Brazilian population [26]. Last, the third level encompassed the delivery source payment (public or private) and parity, stratified in primiparous or multiparous women.

### Statistical analyses

According to the literature, women who have a vaginal delivery present a higher probability of breastfeeding their newborn compared to those who have a c-Sect [21]. Therefore, we stratified all analyses by type of delivery.

First, we performed a descriptive analysis of socioeconomic and demographic characteristics. Hereafter, we described the prevalence of antenatal and postpartum depression and the prevalence of each item that composes the variable obstetric violence.

To evaluate the mediating effect of postpartum depression on the relationship between obstetric violence and breastfeeding, we performed structural equation models using Mplus 8.0. Obstetric violence was a continuous latent variable. We deemed a factor loading greater than 0.5 along with a *p*-value less than 0.05 indicative of a good correlation between the observed variable and the construct of interest [27]. This analysis employed weighted least squares mean, and variance adjusted (WLSMV) estimation, adopting theta parameterization. Considering the loss of information in some variables, we applied the full information method, encompassing the imputation of missing values.

We calculated modification indices using the MODINDICES command to obtain suggestions for changes to our initial hypotheses. Whenever proposed modifications (with modification rates greater than 10) were considered theoretically plausible, a new model was developed. In all analyses, we considered a path significant when the *p*-value was less than or equal to 0.05 [27].

We evaluated model fit employing the root mean square error of approximation (RMSEA), comparative fit index (CFI), and Tucker–Lewis index (TLI). The RMSEA compensates for model complexity by considering the adjustment given the number of parameters involved (i.e., degrees of freedom); values ​​less than 0.06 indicate a good fit. RMSEA confidence limits greater than 90% and below 0.08 indicated a good fit. We inferred adequate model fit from CFI and TLI above 0.95 [28, 29]. Both the CFI and RMSEA are sensitive to the lack of model specification and are affected only slightly by sample size.

### Ethics

This study was approved by the Research Ethics Committee of the School of Physical Education of the Federal University of Pelotas (CAAE 26746414.5.0000.5313) on February 5, 2014. All women provided written informed consent.

## Results

Table 1 provides an overview of the study population characteristics. Approximately two-thirds of the women (65.5%) gave birth by c-section. In both groups, nearly 50% were aged between 20 and 29 years. Among the women who experienced vaginal delivery, 65% self-identified as white, contrasting with 75% among those who had a c-section. The proportion of women cohabiting with a partner hovered approximately 80% for both groups. Among the c-section group, 40% had attained 12 or more years of education, whereas among the vaginal delivery group, this proportion was 14%. Regarding economic position, the proportions of women belonging to the A group (richest) and D/E group (poorest) were 14.8% and 13.1%, respectively, in the C-section group, while these proportions were 3% and 28.5% among the vaginal birth group. About 94% of the vaginal delivery group had the childbirth payment made by the public sector, while in the c-section group, this proportion was 51%. Primiparous women constituted 43% and 55% of the vaginal and C-section delivery groups, respectively. The prevalence of antenatal depression was higher among women who had a vaginal birth (33%) compared to those who had a c-Sect. (27%), as well as the prevalence of postpartum depression (13% vs. 9%). All four items incorporated in the latent variable obstetric violence presented higher prevalence among the vaginal delivery group. However, only verbal abuse and undesired/non consented procedures presented statistically significant differences. The proportion of women who attempted breastfeeding in the maternity ward was similar for both groups. The average breastfeeding duration was slightly longer among women who had c-section delivery (4.3% vs. 3.9%).

**Table 1:** Descriptive characteristics of women stratified by type of delivery, Pelotas birth cohort 2015

Variables	Vaginal delivery (*n* = 1,278)		C-section (*n* = 2,319)		*p* value
	%	IC95%	%	IC95%	
**Women characteristics**					
Age (years)					
19 or less	21.9	19.7–24.2	10.5	9.3–11.8	< 0.001
20 to 29	49.5	47.0–52.3	47.1	45.1–49.2	
30 to 39	26.8	24.4–29.3	39.0	37.0–41.0	
40 or more	1.8	1.2–2.7	3.3	2.7–4.2	
Ethnicity/skin colour					
White	64.5	61.9–67.1	75.2	73.4–76.9	< 0.001
Non-white	53.5	32.9–38.1	24.8	23.1–26.6	
Marital status					
Living with a partner	80.0	77.2–82.4	85.0	83.2–86.5	0.001
Living without a partner	20.0	17.6–22.8	15.0	13.5–16.7	
Socioeconomic position (ABEP)*					
A (richest)	3.0	2.2–4.1	14.8	13.4–16.3	< 0.001
B	11.4	9.7–13.3	25.1	23.4–30.0	
C	57.1	54.3–59.8	46.9	44.9–49.0	
D/E (poorest)	28.5	26.0 1 31.1	13.1	11.8–14.6	
Educational level (completed years)					
0 to 4	14.2	12.4–16.3	5.4	4.5–6.3	< 0.001
5 to 8	34.4	31.9–37.1	20.3	18.7–22.1	
9 to 11	36.9	34.3–39-6	33.6	31.7–35.5	
12 or more	14.4	12.6–16.4	40.7	38.8–42.8	
Type of payment for delivery					
Private	5.7	4.5–7.1	48.7	46.7–50.8	< 0.001
Public	94.3	92.9–95.5	51.3	49.2–53.3	
Parity					
Primiparous	42.6	40.01–45.4	54.6	52.6–56.6	< 0.001
Multiparous	57.4	54.6–60.0	45.4	43.4–47.4	
**Depression**					
Antenatal depression (EPDS ≥ 10)	33.1	30.1–36.2	27.1	25.1–29.3	0.001
Postpartum depression (EPDS ≥ 13)	12.7	10.9–14.6	9.1	8.0–10.4	0.001
**Obstetric violence**					
Verbal abuse	11.4	9.7–13.3	8.2	7.1–9.4	0.002
Physical abuse	4.8	3.8–6.2	3.9	3.2–4.8	0.195
Negligence/denial of care	6.6	5.3–8.1	5.6	4.7–6.6	0.216
Undesired/non consented procedures	8.8	7.3–10.5	3.9	3.2–4.8	< 0.001
**Breastfeeding**					
Attempt to breastfeeding in the maternity	96.5	95.3–97.4	97.5	96.8–98.1	0.080
Average duration in months of breastfeeding	3.88	3.67–4.10	4.28	4.11–4.45	0.005

*ABEP - Brazilian Economic Classification Criteria

Table 2 shows the findings of the structural equation analyses. The model presented a good fit (RMSEA: 0.010, CFI: 0.995, and TLI: 0.988). Women who suffered obstetric violence were more likely to interrupt breastfeeding earlier than those who did not (direct effect). This effect was observed only among women who had vaginal delivery (-0.224; *p* value: 0.017). No direct effect of obstetric violence on breastfeeding was found among women who had c-section deliveries. The mediation effect of postpartum depression on the relationship between obstetric violence and breastfeeding (indirect effect) was not demonstrated. The total effect (direct + indirect effect) among women who had vaginal delivery was − 0.223, *p* value: 0.016. The total effect for women who had c-sections did not attain statistical significance. Obstetric violence did not present a direct effect on the attempt to breastfeed in the maternity for either group.

**Table 2:** Standardized coefficients, standard error, and *P* value of the direct and indirect effect of obstetric violence on breastfeeding, Pelotas birth cohort 2015

VARIABLES	Vaginal delivery			C-section delivery		
	Standardized coefficient	Standard error	*p* value	Standardized coefficient	Standard error	*p* value
**Latent variable**						
Verbal abuse	0.795	0.054	< 0.001	0.826	0.063	< 0.001
Physical abuse	0.870	0.062	< 0.001	0.573	0.076	< 0.001
Negligence/Denial of care	0.501	0.080	< 0.001	0.501	0.069	< 0.001
Undesired/non-consented procedures	0.755	0.055	< 0.001	0.649	0.069	< 0.001
**Direct effect of each variable included in the model**						
**Obstetric violence (latent variable)**						
Type of payment for delivery	0.476	0.197	0.016	0.567	0.103	< 0.001
Parity	-0.255	0.145	0.079	-0.124	0.079	0.114
Antenatal depression	0.137	0.085	0.110	0.320	0.067	< 0.001
Age	0.190	0.133	0.153	0.004	0.061	0.951
Ethnicity	-0.084	0.083	0.307	-0.061	0.048	0.209
Educational level	0.166	0.117	0.156	0.186	0.071	0.009
Marital status	-0.115	0.073	0.116	-0.014	0.044	0.754
Socioeconomic position	-0.140	0.085	0.100	-0.210	0.069	0.002
Postpartum depression	0.074	0.103	0.469	0.000	0.098	0.997
Breastfeeding in the maternity	-0.147	0.160	0.361	-0.027	0.124	0.827
Breastfeeding duration	-0.224	0.094	0.017	0.016	0.077	0.835
**Breastfeeding duration**						
Breastfeeding in the maternity	0.027	0.102	0.788	0.026	0.100	0.798
Postpartum depression	0.063	0.130	0.625	0.058	0.091	0.526
Type of payment for delivery	0.110	0.209	0.597	-0.005	0.095	0.958
Parity	-0.073	0.144	0.615	0.088	0.060	0.144
Postpartum depression	-0.057	0.107	0.592	-0.157	0.091	0.084
Age	0.080	0.135	0.555	0.009	0.050	0.850
Ethnicity	0.062	0.072	0.391	0.050	0.037	0.181
Educational level	0.053	0.102	0.605	0.131	0.058	0.023
Marital status	-0.058	0.060	0.332	0.021	0.038	0.588
Socioeconomic position	-0.178	0.081	0.028	-0.066	0.056	0.235
**Breastfeeding in the maternity**						
Type of payment for delivery	0.074	0.442	0.867	-0.046	0.148	0.754
Parity	0.445	0.190	0.019	0.108	0.107	0.312
Antenatal depression	-0.171	0.117	0.145	-0.115	0.100	0.252
Age	-0.310	0.232	0.182	-0.078	0.091	0.390
Ethnicity	0.042	0.135	0.755	0.036	0.069	0.602
Educational level	0.035	0.177	0.842	0.138	0.092	0.133
Marital status	-0.004	0.098	0.964	0.002	0.071	0.982
Socioeconomic position	-0.214	0.132	0.104	-0.150	0.099	0.129
**Postpartum depression**						
Type of payment for delivery	0.172	0.304	0.572	0.035	0.119	0.769
Parity	0.073	0.136	0.590	0.038	0.071	0.592
Antenatal depression	0.566	0.065	< 0.001	0.602	0.062	< 0.001
Age	0.080	0.139	0.568	0.002	0.058	0.978
Ethnicity	-0.062	0.088	0.481	0.031	0.043	0.461
Educational level	0.075	0.136	0.584	0.075	0.069	0.273
Marital status	0.030	0.060	0.623	0.068	0.038	0.075
Socioeconomic position	0.052	0.102	0.611	0.024	0.070	0.730
**Type of payment for delivery**						
Parity	0.024	0.117	0.835	0.142	0.037	< 0.001
Antenatal depression	0.090	0.074	0.225	0.041	0.036	0.262
Age	-0.278	0.103	0.007	-0.126	0.032	< 0.001
Ethnicity	0.221	0.070	0.002	0.092	0.025	< 0.001
Educational level	-0.371	0.078	< 0.001	-0.348	0.030	< 0.001
Marital status	0.041	0.073	0.569	0.027	0.023	0.241
Socioeconomic position	0.246	0.076	0.001	0.379	0.030	< 0.001
**Parity**						
Antenatal depression	0.156	0.047	0.001	0.182	0.040	< 0.001
Age	0.705	0.025	< 0.001	0.514	0.025	< 0.001
Ethnicity	-0.069	0.033	0.036	-0.032	0.028	0.246
Educational level	-0.289	0.041	< 0.001	-0.340	0.032	< 0.001
Marital status	-0.170	0.032	< 0.001	-0.093	0.028	0.001
Socioeconomic position	0.117	0.041	0.004	-0.001	-0.019	0.985
**Antenatal depression**						
Age	0.054	0.044	0.212	0.020	0.034	0.563
Ethnicity	0.050	0.043	0.242	0.098	0.031	0.002
Educational level	-0.351	0.044	< 0.001	-0.244	0.035	< 0.001
Marital status	0.069	0.043	0.107	0.051	0.031	0.101
Socioeconomic position	0.033	0.046	0.480	0.140	0.038	< 0.001
**Indirect effect of the obstetric violence on breastfeeding duration**						
Obstetric violence → Postpartum depression → Breastfeeding duration	0.005	0.012	0.700	0.000	0.006	0.997
**Total Effect (direct + indirect effect) of the obstetric violence on breastfeeding duration**						
Obstetric violence → Postpartum depression → Breastfeeding duration	-0.223	0.093	0.016	0.015	0.076	0.840

## Discussion

This study has shown that experiencing obstetric violence negatively impacts breastfeeding duration. This effect varied depending on the type of delivery and was evidenced only among women who had vaginal delivery. We were unable to demonstrate an indirect effect mediated through postpartum depression. Our analyses did not identify any effect of exposure to obstetric violence on the attempt to breastfeed in the maternity ward.

The relationship between obstetric violence and breastfeeding has not received scientific attention so far. To the best of our knowledge, there is only one study on the topic, also conducted in Brazil [19]. The results showed that women who experienced obstetric violence had lower rates of exclusive breastfeeding when they were discharged from the maternity ward. This effect was observed for both vaginal and c-section delivery, with a stronger impact among those who had a vaginal delivery (coefficient: −0.267 vs. -0.105). In contrast, we did not identify any significant effect of obstetric violence on the attempt to breastfeed in the maternity ward. We hypothesized that the discrepancy may be attributed to differences in the questions posed to the women. While the Leite et al. (2023) study inquired whether the newborn was discharged from the maternity ward being breastfeeding exclusively, our study asked if the women had put the newborn on their breast, which can be considered a proxy of breastfeeding the newborn in the maternity ward. Therefore, in our study a positive response to this question leaves open the possibility that breastfeeding was not done exclusively, as women who used supplemental formula feeding may also have answered in the affirmative.

Leite et al. (2023) also demonstrated that obstetric violence presents an indirect effect on women’s capacity to breastfeed up to six months after childbirth through breastfeeding initiation in the maternity ward with a stronger effect in the vaginal delivery group compared to the C-section group (coefficient: −0.134 vs. −0.055). This result is consistent with our study for the vaginal delivery group. Otherwise, Leite et al. (2023) did not identify an effect of obstetric violence on the c-section group. This disparity might be because women who had a c-section suffer less severe forms and present a lower prevalence of obstetric violence [15, 16], especially those who did not go into labour before the c-Sect [16]. This may indicate that women who have a vaginal birth or who go into labour before a caesarean section suffer more obstetric violence due to the greater demand for care from health professionals and longer exposure time, specially concerning to verbal and physically abuse and negligence or denial of care. In the case of women who have already gone into labour but subsequently require a C-section, its important to consider that the obstetric violence could be related in greater extension to undesired or non-consented procedures, since the difficulty in stablishing fast and effective communication among healthcare professionals and women in case of emergencies or complex medical situations.

The literature suggests a negative impact of postpartum depression on breastfeeding [15, 17, 24, 30]. However, in contrast to our initial hypothesis, we were unable to observe an effect as a mediator in the relationship between obstetric violence and breastfeeding. We bring to discussion four potential explanations for this result: (1) the magnitude of the mediator/indirect pathway effect is truly small, and the sample size did not provide sufficient statistical power to detect a true effect; (2) the prevalence of postpartum depression was not high enough, and we had no statistical power to identify the effect; (3) the construct of obstetric violence may be affected by bias due to misclassification, attenuating differences and diminishing the observed effect; and (4) postpartum depression is not in fact a mediator.

It is also important to consider additional factors that may contribute to the lack of effect of postpartum depression as a mediator. The challenge in establishing successful breastfeeding typically arises in the initial days post-childbirth; once established, breastfeeding often continues successfully. Postpartum depression was assessed three months after birth. By this time, breastfeeding may have already been well established, potentially explaining the absence of a mediating effect.

Our study is the first to incorporate postpartum depression as a possible mediator. Moreover, there are other mental disorders – not included in this study – that may mediate the relationship between obstetric violence and breastfeeding, such as posttraumatic stress disorder or generalized anxiety disorder. Consequently, further research is needed to elucidate the potential underlying mechanisms inherent in the relationship between obstetric violence and breastfeeding.

To understand the plausibility of the possible relationship between obstetric violence and breastfeeding, some hypotheses deserve to be highlighted. First, the literature suggests the positive role of health professionals in stimulating, supporting, and managing breastfeeding, especially during the critical hours after childbirth [31]. Suffering violence from professionals responsible for stimulating and supporting breastfeeding could disrupt the trust relation between women and professionals and impair the early initiation and maintenance of breastfeeding. In addition, the health team needs to implement evidence-based protocols to improve breastfeeding, such as keeping the mother and baby together in the golden hour, skin-to-skin contact, discouraging the use of pacifiers, and most importantly, offering support to mothers who have not yet established breastfeeding [21, 31, 32].

Furthermore, it is well established that individuals exposed to situations of stress and suffering, such as the experience of violence, present a cascade of hormonal changes, including the inhibition or cessation of oxytocin production [32], and behavioral alterations characterized by negative emotions [33], which can negatively impact breastfeeding. Although not addressing the type of violence under investigation, the literature has shown that women victims of violence by intimate partners during pregnancy are less likely to intend to breastfeed, start breastfeeding, sustain it for an adequate time, and present a greater risk of prematurely discontinuing exclusive breastfeeding [34, 35].

Finally, another pathway to explain the relationship between obstetric violence and breastfeeding is through postpartum depression. First, postpartum depression damages the establishment of a connection between women and newborns, which is a fundamental factor for the success of breastfeeding. In addition, people living with depression are more likely to consume alcohol and other drugs. Thus, the discontinuation of breastfeeding could be an attempt by the mother to protect the baby from harmful substances such as breast milk. This hypothesis was weakened by our results once the indirect effect of postpartum depression had not been observed. Even so, given the limited evidence on the subject, it is premature to discard this hypothesis.

Our study is affected by limitations. It is important to address that many women in Brazil are unaware of what constitutes obstetric violence. Additionally, the interventionist culture of childbirth is still normalized in most Brazilian institutions. These structural issues could introduce an underreporting bias in the prevalence of obstetric violence. To mitigate this possibility, we focused not on the women’s perceptions but on specific acts (e.g., cursing, humiliation, beating, pushing), as well as denial of care and procedures conducted against the woman’s wishes. The main limitation concerns the restricted capacity of the instrument to adequately measure obstetric violence occurrence, which could negatively affect the identification of any relationship and pathways. Another limitation is the absence of information regarding the successful initiation of breastfeeding in the maternity setting. Last, the low prevalence of postpartum depression allied to the stratification of analyses by type of delivery may have precluded us from demonstrating their effect as a mediator of the association between obstetric violence and breastfeeding due to a lack of statistical power.

The main strength of our study is its innovative characteristics. This is the first study to investigate the role of postpartum depression as a mediator of the relationship between obstetric violence and breastfeeding and the second one to evaluate the effect of this type of violence on breastfeeding. Furthermore, the study design allows us safely to establish the temporality of the events. Last, the inclusion of information on antenatal depression collected during pregnancy and the low refusal rate of follow-up contribute to the study’s robustness.

In conclusion, the findings showed that obstetric violence affects breastfeeding among women who had vaginal delivery and that postpartum depression was not a mediator of this relation. Further studies focused on this relationship are necessary in other contexts and populations. Furthermore, our study reinforces the importance of developing instruments with robust psychometric properties to assess obstetric violence occurrence. Our study is a significant contribution to the understanding of a topic whose evidence is truly rare and represents an important advance in unraveling the causal network that explains the pathways connecting obstetric violence to breastfeeding.

## Data Availability

The datasets generated and/or analysed during the current study are not publicly, but de-identified data could be available from the corresponding author upon reasonable request and approval from the 2015 Pelotas Birth Cohort Coordinators.

## Abbreviations

WHO: World Health Organization
HIV: Human Immunodeficiency Virus
EPDS: Edinburgh Postnatal Depression Scale
ABEP: Brazilian Economic Classification Criteria
WLSMV: Weighted Least Squares Mean and Variance Adjusted
RMSEA: Root Mean Square Error of Approximation
CFI: Comparative Fit Index
TLI: Tucker-Lewis Index
